# Supplementary material for: Intercellular signaling stabilizes single-cell level phenotypic transitions and accelerates the reestablishment of equilibrium of heterogeneous cancer cell populations
Source: bioRxiv. 2025 Jan 4:2025.01.03.631250. Preprint. [Version 1] doi: 10.1101/2025.01.03.631250 (PMC11722408; doi:10.1101/2025.01.03.631250)
Supplement: Supplementary file 1 [file media-1.pdf]

Supplementary Figure 1

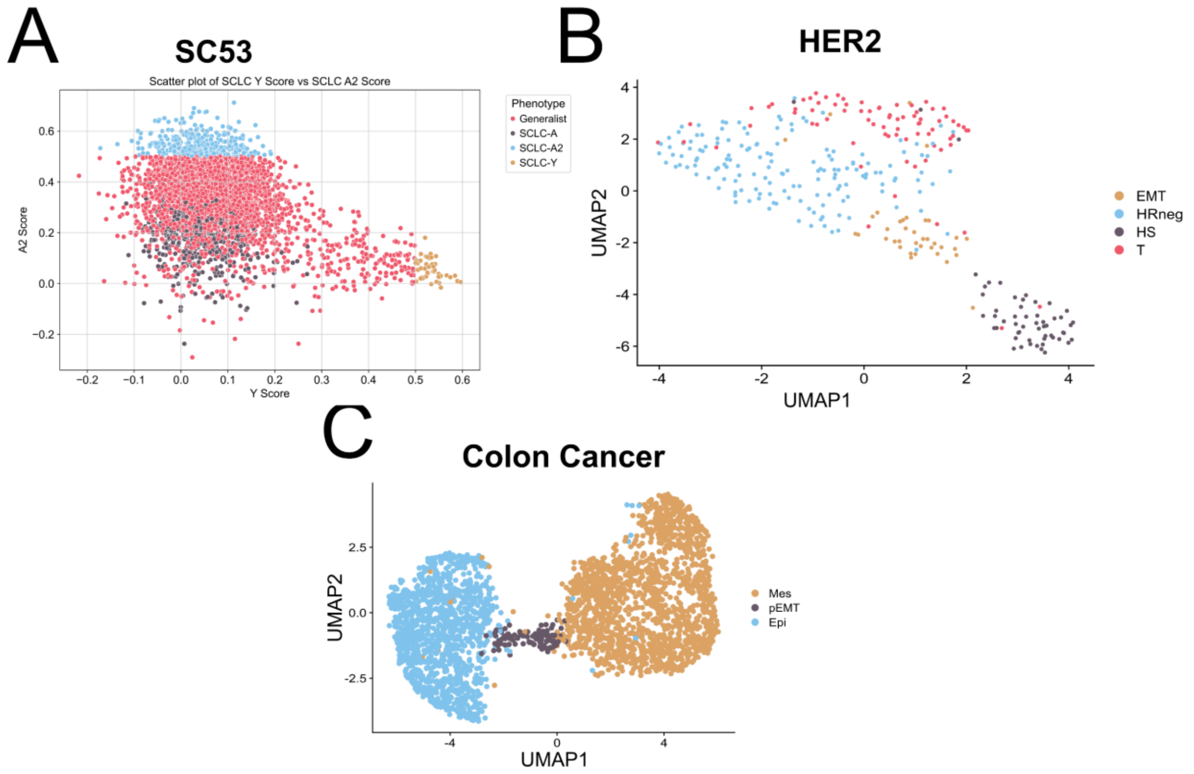

Supplementary Figure 1: ***Plots displaying the different cell types/cell-states present in SC53, HER2 and colon cancer data.*** *A) Cell types present in the CTX SC53 dataset. Cell types were determined by archetype analysis. Archetype analysis scores for the Y and A2 subtype are used to plot the different cells. B) cell types from the t039232 sample of the HER2 dataset. Cell types were determined based on the enriched expression of cell type markers from the terminal branches of trajectory analysis. C) Cell types from the colon cancer datasets (HCT1116 cell line). Cell types are characterized by Epcam status from FACS and scRNA-seq analysis*

Supplementary Figure 2

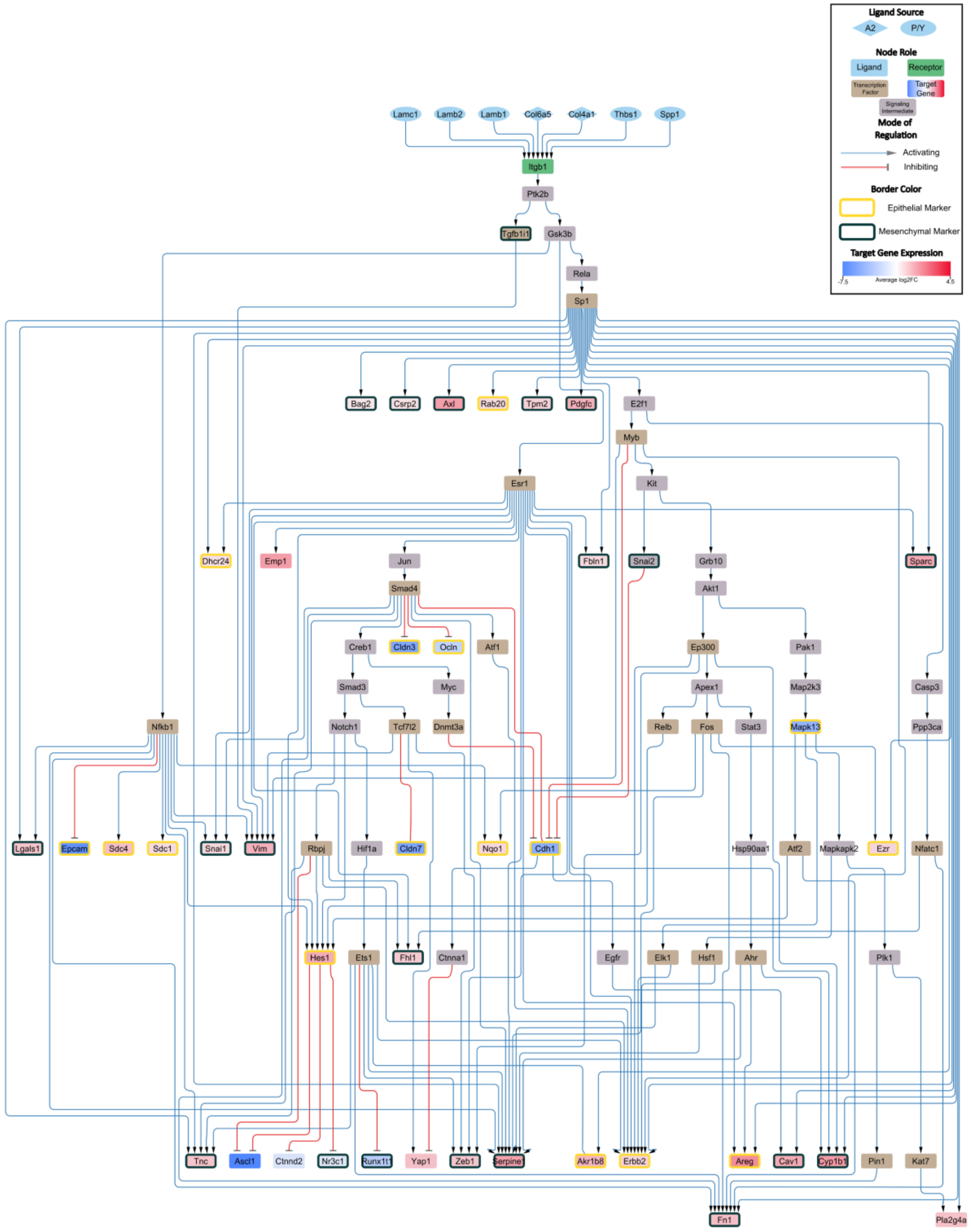

*Supplementary Figure 2: **P/Y cells at timepoint 11 utilize signals from NE and non-NE cell types to reinforce mesenchymal phenotype**. The node color represents the the role of the node/gene in the network, with the target genes colored based on log-fold change value from dark blue to red. Yellow borders around nodes represent epithelial markers and black borders are mesenchymal markers. Line width from ligands (light blue nodes) to receptors (green nodes) represent inferred interaction strength from CellChat.*

Supplementary Figure 3

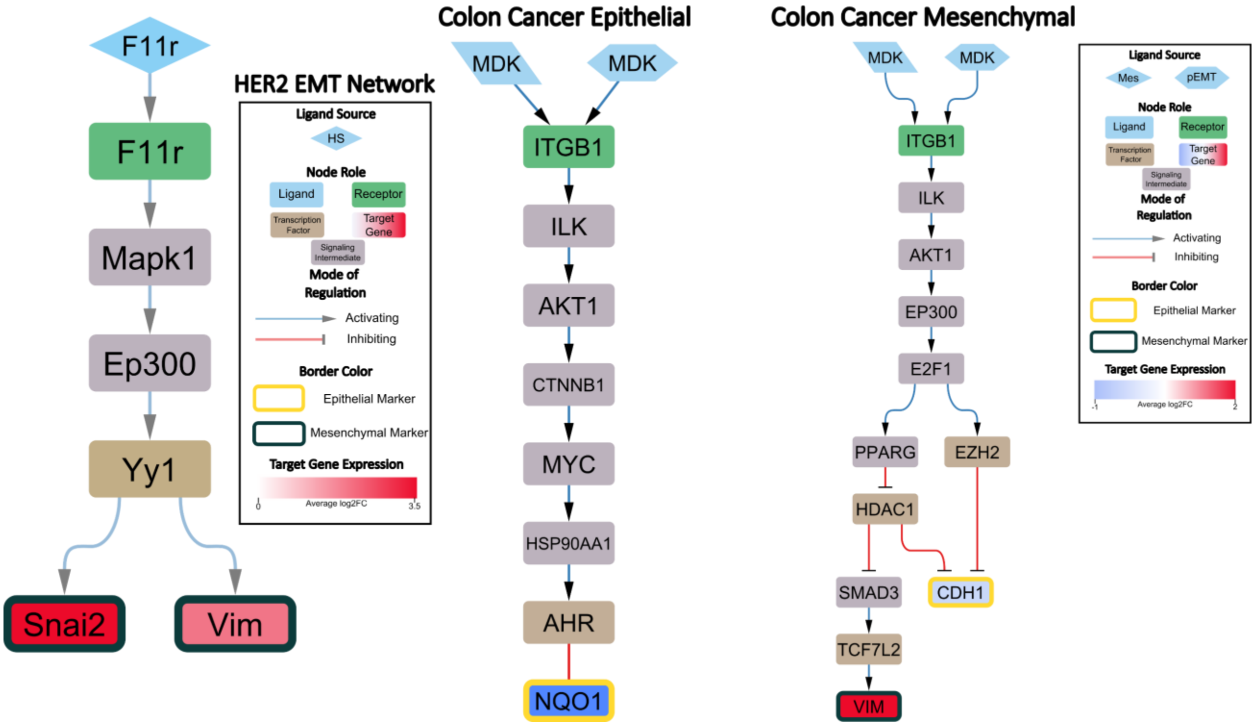

Supplementary Figure 3: ***Signaling networks created from multiscale inference approach for HER2 and colon cancer data.*** *A) The mesenchymal (EMT) cells in the HER2 dataset utilize signals from the more epithelial-like cells to maintain its phenotype. B) Signaling network for the epithelial cells in the colon cancer dataset. C) Inferred mesenchymal cell type signaling network in the colon cancer dataset.*

Supplementary Figure 4

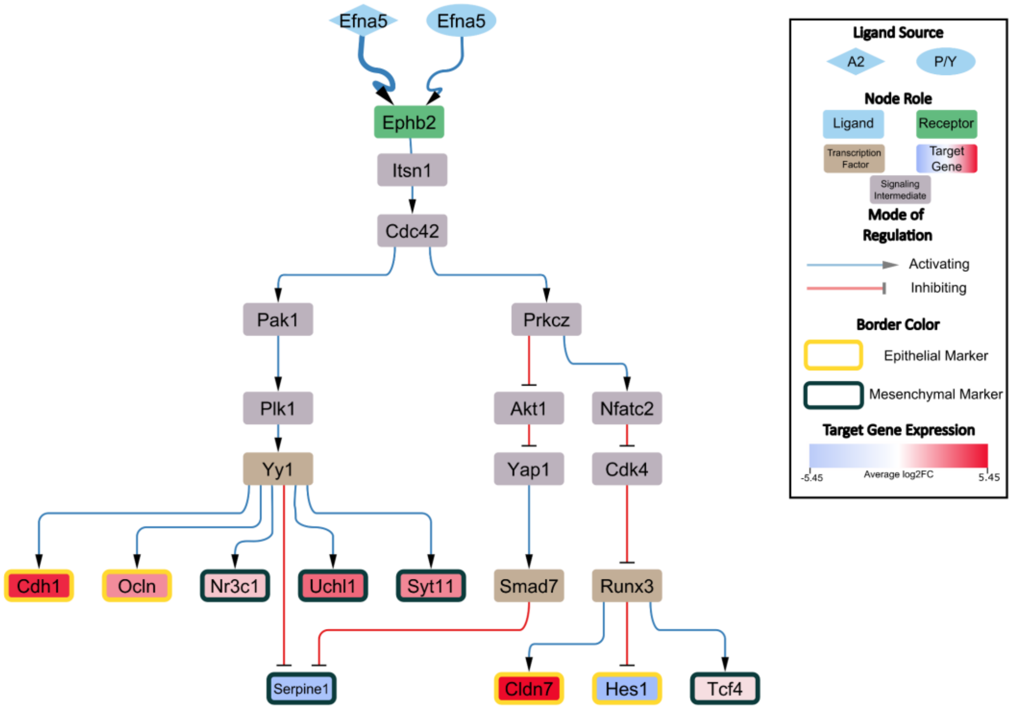

*Supplementary Figure 4: **The A2 cells in RPM data use signals from both NE and non-NE cells to maintain its epithelial phenotype.** Ligands are from A2 and P/Y cells.*

Supplementary Figure 5

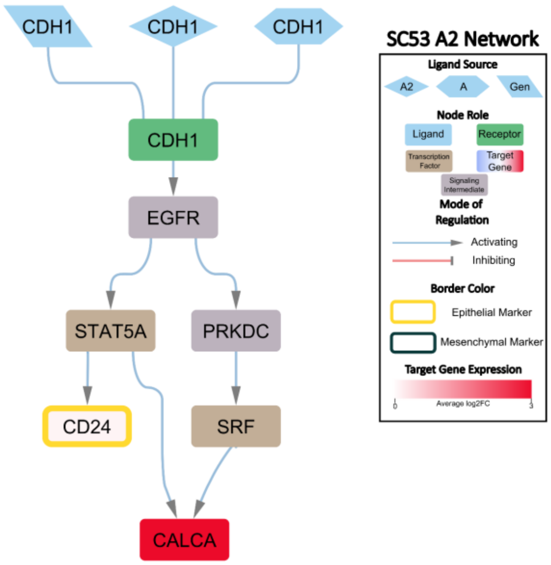

Supplementary Figure 5: ***Interactions between A2 cells help to reinforce the epithelial phenotype in SC53.*** *Ligands are from A2, A and Generalist cells. CALCA is an A2 marker*
